# Roles and inhibitors of FAK in cancer: current advances and future directions

**DOI:** 10.3389/fphar.2024.1274209

**Published:** 2024-02-12

**Authors:** Hui-Hui Hu, Sai-Qi Wang, Hai-Li Shang, Hui-Fang Lv, Bei-Bei Chen, She-Gan Gao, Xiao-Bing Chen

**Affiliations:** ^1^ Department of Oncology, The Affiliated Cancer Hospital of Zhengzhou University and Henan Cancer Hospital, Henan Engineering Research Center of Precision Therapy of Gastrointestinal Cancer and Zhengzhou Key Laboratory for Precision Therapy of Gastrointestinal Cancer, Zhengzhou, China; ^2^ State Key Laboratory of Esophageal Cancer Prevention & Treatment, Zhengzhou University, Zhengzhou, China; ^3^ Henan Key Laboratory of Microbiome and Esophageal Cancer Prevention and Treatment, Henan Key Laboratory of Cancer Epigenetics, Cancer Hospital, The First Affiliated Hospital of Henan University of Science and Technology, Luoyang, China

**Keywords:** focal adhesion kinase, signal pathway, drug resistance, immune microenvironment, inhibitor, IN10018, defactinib

## Abstract

Focal adhesion kinase (FAK) is a non-receptor tyrosine kinase that exhibits high expression in various tumors and is associated with a poor prognosis. FAK activation promotes tumor growth, invasion, metastasis, and angiogenesis via both kinase-dependent and kinase-independent pathways. Moreover, FAK is crucial for sustaining the tumor microenvironment. The inhibition of FAK impedes tumorigenesis, metastasis, and drug resistance in cancer. Therefore, developing targeted inhibitors against FAK presents a promising therapeutic strategy. To date, numerous FAK inhibitors, including IN10018, defactinib, GSK2256098, conteltinib, and APG-2449, have been developed, which have demonstrated positive anti-tumor effects in preclinical studies and are undergoing clinical trials for several types of tumors. Moreover, many novel FAK inhibitors are currently in preclinical studies to advance targeted therapy for tumors with aberrantly activated FAK. The benefits of FAK degraders, especially in terms of their scaffold function, are increasingly evident, holding promising potential for future clinical exploration and breakthroughs. This review aims to clarify FAK’s role in cancer, offering a comprehensive overview of the current status and future prospects of FAK-targeted therapy and combination approaches. The goal is to provide valuable insights for advancing anti-cancer treatment strategies.

## 1 Introduction

**FIGURE 1:**
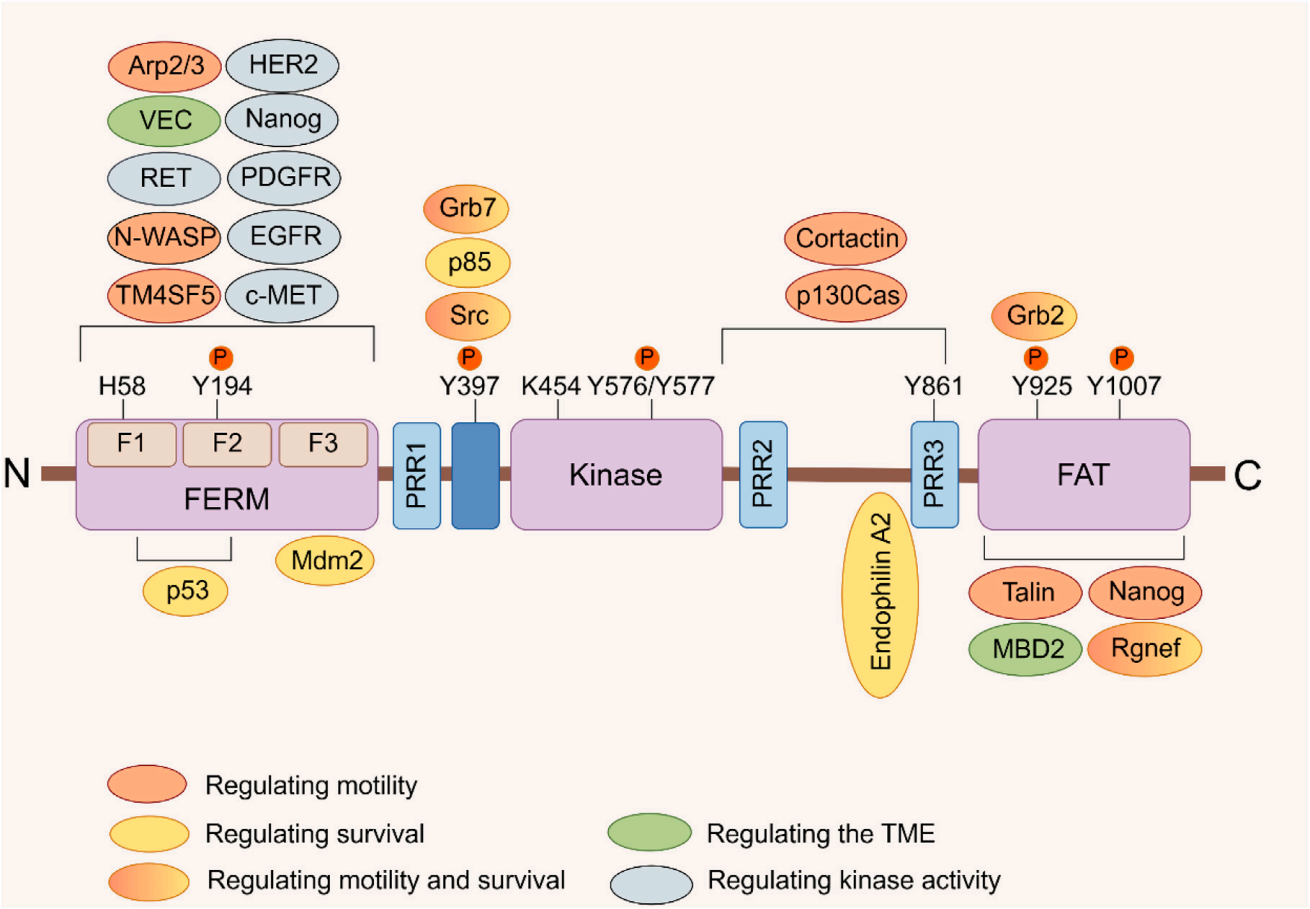
FAK domain structure. FAK consists of a central kinase domain flanked by a FERM homology domain on the N-terminal side and a C-terminal FAT domain. Both the terminal domains are separated from the kinase domain by a linker region containing proline-rich regions (PRR). Important tyrosine (Y) phosphorylation (P) sites are indicated; Y397, K454, and H58 play crucial roles in FAK activation. FAK binding partners are displayed at their interaction sites within FAK. The color signifies the function of FAK interacting proteins, which facilitate diverse activities of cancer cells by interacting with FAK (Sulzmaier et al., 2014).

Focal adhesion kinase (FAK), also known as PTK2, is a non-receptor tyrosine kinase encoded by the PTK2 gene. It plays a critical role in signal transduction mediated by both growth factor receptors and integrins (Kornberg et al., 1992). Upon activation by various extracellular signals received through transmembrane receptors on the cell surface, FAK aggregates in a focal adhesion manner in the cytoplasmic membrane. Autophosphorylation at tyrosine residue 397 (Tyr397), leading to FAK’s activation and initiation of downstream signaling cascades. This process involves a conformational change exposing the phosphorylation site through lipid binding (Acebron et al., 2020; Le Coq et al., 2022). The FAK protein consists of three distinct domains: an N-terminal 4.1 ezrin-radixin-moesin domain (FERM), a central kinase domain, and a C-terminal focal adhesion targeting (FAT) domain (Figure 1) (Lim et al., 2008). Through its FERM domain, FAK acts as an intracellular scaffold facilitating the interconnection of multiple oncogenic signaling pathways via diverse protein-protein interactions. FAK positively regulates tumor progression by promoting cell proliferation, survival, intracellular signaling, and angiogenesis. It also contributes to maintaining the stability of the tumor microenvironment (TME) (Sulzmaier et al., 2014; Dawson et al., 2021). At the adhesion sites of cells with the extracellular matrix (ECM), FAK enhances cellular dynamics, migration, and invasion capabilities by participating in the formation of molecular complexes within the actin and adhesion regulation network (Sulzmaier et al., 2014; Schoenherr et al., 2017; Tapial et al., 2020).

FAK stands out as a key player in cancer pathogenesis, being abnormally activated across various cancer types. Its expression level not only is inversely linked to patient survival but also positions FAK as a pivotal target for thwarting tumor progression and curbing recurrence (Zeng et al., 2016; Kanteti et al., 2018; Aboubakar et al., 2019a; Aboubakar et al., 2019b; Qiao et al., 2020; Wei et al., 2021; Roy-Luzarraga et al., 2022). Rigorous meta-analyses underscore the significance of heightened FAK expression in predicting unfavorable overall survival (OS) outcomes in a spectrum of solid tumors. These include gastric cancer, ovarian cancer, endometrial cancer, glioma, breast cancer, and squamous cell carcinoma (Zeng et al., 2016; Qiao et al., 2020). Compared to non-small cell lung cancer (NSCLC), small-cell lung cancer exhibits a higher degree of malignancy and is more prone to early-stage metastasis. It is worth noting that the expression of FAK in small-cell lung cancer is significantly elevated compared to other types of lung cancer, which implying its potential association with the degree of malignancy as well as invasion and metastatic capabilities (Aboubakar et al., 2019b).

As a paralogous homolog of FAK, proline-rich tyrosine kinase 2 (PYK2) displays a similar multi-domain organization and protein binding sites to FAK, forming a subfamily of adhesion kinases together with FAK that is crucial in regulating signaling networks involved in tumor growth and metastasis (Sulzmaier et al., 2014; Naser et al., 2018). Unlike FAK, PYK2 does not localize to focal adhesions, relying instead on intracellular calcium mobilization for activation (Avraham et al., 2000). Moreover, inhibiting FAK can induce an increase in the expression or phosphorylation of PYK2 in cancer cells (Fan and Guan, 2011). The concurrent targeting of both FAK and PYK2 is believed to confer a more advantageous anti-cancer effect. FAK kinase inhibitors are typically classified as either FAK specific inhibitors or dual FAK/PYK2 inhibitors (Berger et al., 2021). However, there is currently no clinical evidence demonstrating specific differences in therapeutic efficacy between the use of FAK or dual FAK/PYK2 kinase inhibitors (Dawson et al., 2021). Multikinase inhibitors containing FAK, such as Conteltinib and APG-244, have exhibited significant efficacy in specific tumor types and are currently undergoing clinical investigation (Xing et al., 2022; Zhao et al., 2022). Despite the promising anti-tumor activity demonstrated in preclinical studies, the clinical efficacy of FAK inhibitors as a monotherapy for anticancer treatment remains limited. This limitation stems from the dual role of FAK, acting both as a kinase and a protein scaffold, which mediates drug resistance through crosstalk with specific signaling pathways in the network (Gerber et al., 2020). As adjuvant therapy, the combination of FAK inhibitors with conventional chemotherapy agents as shown enhanced anti-tumor potential (Dawson et al., 2021). Consequently, the exploration of combining FAK inhibitors with chemotherapy, radiotherapy or immunotherapy has become as a focal point of research in recent years.

This review critically delves into the role of FAK in regulating tumor cell signal transduction, diverse cellular activities, the immune microenvironment, and drug resistance. Additionally, it explores the current research progress on FAK inhibitors in anticancer therapy, aiming to provide new insights for the future development and application of FAK inhibitors in the field.

## 2 Role of FAK in different signaling pathways

### 2.1 PI3K/AKT signaling pathway

**FIGURE 2:**
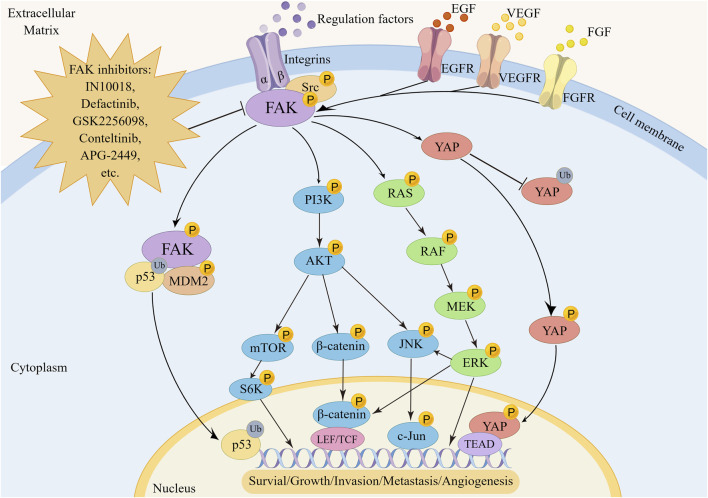
The regulatory mechanism of FAK in tumorigenesis, metastasis and angiogenesis. FAK promotes oncogenesis by activating transcription factors via the p53, YAP, RAS/RAF/MEK/ERK, PI3K/AKT, and downstream pathways including mTOR, β-catenin, or JNK.

FAK plays a pivotal role in regulating the survival and development of tumor cells through mediating multiple signaling pathways (Figure 2). One key regulatory pathway for FAK is the PI3K/AKT signaling pathway, which mediates various cellular functions, such as proliferation, survival, migration, invasion, and metastasis (Fresno et al., 2004). When activated, FAK forms complexes with PI3K, boosting PI3K activation and generating more PIP3, initiating downstream pathway signaling. In uveal melanoma (UM), characterized by activating mutations in GNAQ/GNA11, encoding the Gαq protein, FAK has been identified as a central mediator of Gαq-driven signaling (Feng et al., 2019; Paradis et al., 2021). Additionally, a whole-genome CRISPR screen has revealed that activation of the PI3K/AKT pathway is not only essential for the survival of human UM cells but also contributes to drug resistance. Specifically, Gαq can upregulate FAK expression and subsequently activate PI3K by binding to its p85 regulatory subunit and inducing tyrosine phosphorylation, thereby initiating downstream signaling cascades associated with cancer promotion (Arang et al., 2023). Targeting FAK effectively blocks this process. As a crucial upstream regulator of the PI3K/AKT pathway, FAK activates PI3K/AKT and its related downstream effectors, including mTOR, β-catenin and p53, promoting cancer progression (Claesson-Welsh and Welsh, 2013; Sulzmaier et al., 2014; Guo et al., 2020). The PI3K/AKT/mTORC1 pathway is a widely targeted therapeutic pathway for various cancer types (Popova and Jucker, 2021; Tewari et al., 2022). Despite the promising clinical activity of mTORC1 inhibitors, their effectiveness in treating breast cancer is limited, with certain patients developing rapid drug resistance. Inhibiting FAK has been shown to enhance the sensitivity of rapamycin resistant tumors to mTORC1 inhibition, revealing an inherent reliance of mTORC1-resistant tumors on FAK (Cuellar-Vite et al., 2022). Combining FAK inhibitors with mTORC1 inhibitors to improve efficacy against drug-resistant cancers appears feasible (Shi et al., 2016; Liao et al., 2022; Qiao et al., 2022; Wang Z et al., 2022; Yang F et al., 2022), although further validation through clinical studies is necessary. In addition, activation of the PI3K/AKT/β-catenin pathway by FAK is crucial for promoting β-catenin nuclear translocation, influencing the transcriptional regulation of tumor cell genesis (Xing et al., 2021; Lee et al., 2023).

### 2.2 P53 signaling pathway

P53, a prominent tumor suppressor gene, plays a crucial role in regulating tumor growth and maintaining the anti-cancer effect properties associated with cell cycle regulation (Hu et al., 2021). The relationship between FAK and p53 is intricate. Studies have demonstrated that p53 directly binds to the PTK2 promoter, inhibiting the transcription of PTK2 (Golubovskaya and Cance, 2010). Various drugs achieve FAK targeting effect by modulating this interaction (Lin et al., 2019; Klungsaeng et al., 2020; Miao et al., 2020; Shen et al., 2022). Furthermore, p53 can indirectly downregulate FAK expression. Wild-type p53 transactivates the transcription of immunoglobulin superfamily 9 (IGSF9), and the resulting IGSF9 interacts with FAK, inhibiting FAK/AKT signal transduction in breast cancer (Li H et al., 2022). Recent research has unveiled that the extracellular domain (ECD) of IGSF9 binds to T cells, inhibiting their proliferation and activation, thereby creating a microenvironment conducive to tumor growth (Liu X et al., 2023a). Consequently, upregulating IGSF9 expression to inhibit FAK function may not be an ideal strategy. Additionally, the impact of FAK as a scaffold protein on p53 can be critical. Studies indicate that nuclear FAK promotes the ubiquitination of p53 in a kinase-independent manner by enhancing MDM2 activity. The inactivation of p53 by FAK requires the binding of FAK FERM F1 leaf and p53 FERM F2 leaf, facilitating the nuclear localization of FAK, while FERM F3 leaf connects with MDM2, mediating proteasomal degradation (Lim et al., 2008; Steinberg et al., 2023). Targeting specifically the FAK FERM domain of FAK using its scaffold function may offer novel therapeutic avenues to counteract this pro-cancer effect (Pomella et al., 2022). However, clinical and preclinical studies have revealed that higher FAK copy number and gene overexpression are associated with worse disease-free survival in patients with mutant-type p53, but not in patients with wild-type p53 (Lakshmanan et al., 2021; Yu et al., 2021; Pifer et al., 2023). This could be attributed to the fact that mutated p53 loses its inhibitory effect on FAK transcription, restoring the positive influence of FAK on the biological behavior of tumor cells. Lakshmanan et al. developed two genetically engineered lung cancer mouse models Kras^G12D/+^; Trp53^R172H/+^; Ad-Cre (KPA) and Kras^G12D/+^; Ad-Cre (KA), revealing the ST6GalNAc-1/MUC5AC axis as a mediator of mutant p53’s regulation of FAK signal transduction. The p53 R175H mutation leads to increased expression of ST6GalNAc-I, promoting glycosylation of MUC5AC and enhancing its interaction with integrin β4. This, in turn, increases phosphorylation of FAK at Y397, ultimately promoting lung cancer invasion and liver metastasis (Lakshmanan et al., 2021).

### 2.3 RAS/RAF/MEK/ERK signaling pathway

Abnormal activation or gene mutation of the RAS/RAF/MEK signaling pathway is a crucial factor in sustaining tumor survival and invasion (Imperial et al., 2019). In recent years, targeting RAS, RAF, or MEK to disrupt pathway transduction has emerged as a promising breakthrough in cancer treatment (Song et al., 2023). Intriguingly, there is a close relationship between FAK expression and the transduction of the RAS/RAF/MEK pathway. FAK promotes the invasion and metastasis of tumor cells by regulating the activation of the RAS/RAF/MEK/ERK signaling pathway (Shao et al., 2022; Yoon et al., 2022). A study has identified a significant correlation between ERK5 activity and FAK expression, as well as Ser910 site phosphorylation in lung cancer and malignant melanoma. ERK5 increases the expression of the transcription factor USF1, which, in turn, upregulates the expression of FAK and activates the FAK signal to promote cell migration (Jiang W et al., 2020). Furthermore, TCGA cancer survival data indicates that lower RNA expression of PTK2 is associated with better survival outcomes in KRAS mutated tumors (Zhang L et al., 2021). This suggests that FAK could potentially serve as an effective biomarker for cancer development induced by abnormal KRAS signaling. Inhibiting the expression of KRAS G12C induces sustained activation of FAK, subsequently restoring the vitality of KRAS G12C mutant tumor cells, including a human CRC cell line (SW837), a human Pancreatic cancer cell line (Mia PaCa-2), and 3 human NSCLC cell lines (NCI-H23, NCI-H1792, and NCI-H2122), through the FAK/YAP pathway (Zhang L et al., 2021). High-throughput transcriptome sequencing of three cell lines from malignant peripheral nerve sheath tumors resistant to MEK inhibitors, revealed that the upregulation of FAK/SRC, leading to the reactivation of the ERK signaling pathway, is a crucial factor in cell resistance. Therefore, the compensatory activation of FAK-related pathways, such as FAK/SRC and FAK/YAP, is a crucial pathway contributing to the ineffectiveness of single targeted therapies for RAS, RAF, or MEK in KRAS mutant tumors. Combining FAK inhibition therapy can potentially restore the sensitivity of drug-resistant cells to MEK or KRAS inhibitors, improving their anti-tumor effect (Gu et al., 2022; Tarin et al., 2023). Based on these preclinical research findings, a phase II clinical trial (NCT04620330) is currently underway (Capelletto et al., 2022) to investigate the true efficacy of the combination therapy of FAK inhibitor defactinib and the RAF/MEK inhibitor VS-6766 for KRAS-mutant NSCLC. FAK holds the potential to serve as a promising therapeutic target for future combination therapy in KRAS mutant tumors.

### 2.4 YAP signaling pathway

The Yes-associated protein (YAP), a primary downstream effector of the Hippo pathway, exhibits dynamic cellular localization, influencing its role as a transcriptional activator. While YAP in the cytoplasm undergoes ubiquitination and degradation, nuclear YAP acts on TEA domain DNA-binding proteins to induce gene transcription (Heng et al., 2021). Phosphorylation intricacies govern YAP’s cytoplasmic-nuclear translocation. Phosphorylation at S127 and S397 suppresses nuclear localization, while Y357 phosphorylation enhances YAP stability and facilitates nuclear translocation, with FAK playing a crucial role in this phosphorylation process (Lachowski et al., 2018; Fard et al., 2023). In cholangiocarcinoma, FAK and activated AKT synergize to induce the YAP oncogene, promoting AKT/Jag1-driven biliary tract cancer occurrence (Song et al., 2021). Interestingly, YAP reciprocally activates FAK, contributing to tumor cell migration (Goto et al., 2020). The YAP-TEAD axis, highlighted by Jie Shen et al., plays a crucial role in inducing FAK activation by targeting platelet-derived growth factor 1 (THBS1) in breast cancer (Shen et al., 2018). The FAK-YAP signaling pathway emerges as a key player in chemotherapy resistance and cancer relapse. Activation of the FAK-YAP cascade, disrupts COL17A1, enabling LGR5+p27+ cancer stem cells (CSCs), to exit dormancy, re-enter the cell cycle, and restore proliferative capacity. Targeting YAP through TEAD inhibitors provides an effective strategy to impede tumor organoid regeneration, offering a novel approach for overcoming cancer recurrence following chemotherapy (Ohta et al., 2022). Furthermore, YAP-FAK contributes significantly to radiotherapy resistance. The focal adhesion component, p130Cas, regulates the YAP-FAK signaling pathway, mediating radiotherapy resistance in NSCLC. p130Cas directly interacts with FAK to regulate YAP activation and nuclear translocation (Li J et al., 2022). Targeting the p130Cas-FAK interaction merges as a potentially cost-effective strategy for overcoming YAP activation-mediated radioresistance in NSCLC.

## 3 Role of FAK in the occurrence and development of tumors

### 3.1 FAK promotes tumor cell survival and proliferation

FAK plays a pivotal role in promoting the survival and proliferation of tumor cells through both kinase-dependent and kinase-independent mechanisms, orchestrating downstream signal transduction. Recent insights highlight FAK’s dual role in phenotype transition, with distinct functions in the cytoplasmic and nuclear compartments. In the cytoplasm, activated FAK initiates survival pathways in a PI3K and MAPK-dependent manner. Simultaneously, within the nucleus, the FERM domain of FAK hinders the activation of p53, thereby preventing inherent cell apoptosis (Del et al., 2022; Ke et al., 2022). Previous studies have delineated various mechanisms through which FAK promotes tumor cell survival and proliferation. These include activating NF-κB to mediate the expression of inhibitor of apoptosis proteins (IAPs), inducing upregulation of cyclin D1 through the ERK pathway activation, and interacting with receptor-interacting protein (RIP) via the death domain kinase to neutralize the pro-apoptotic function of RIP (Chuang et al., 2022). Moreover, FAK contributes to maintaining tumor cell survival by countering anoikis, a form of cell death induced by cell detachment from the ECM. FAK reduces the sensitivity of cancer cells to receptor ligands inducing death by stabilizing the TPL2 protein (Del et al., 2022). Additionally, FAK inhibits cellular senescence, a process crucial for maintaining cell survival (Fard et al., 2023; Steinberg et al., 2023; Tien et al., 2023). Notably, the Sema6C protein, initially recognized as Semaphorin Y, not only forms a complex with tyrosine kinases c-Abl, activating FAK, and leading to the nuclear localization of the YAP transcriptional co-activator. This interaction enables YAP-dependent cancer cells to survive under nutrient deprivation conditions. Inhibition of Sema6C expression reverses these effects, inducing cellular senescence (Fard et al., 2023).

The dysregulation of CDK4/6 in tumor cells is a critical factor in sustaining tumor cell proliferation, making CDK4/6 inhibitors a focal point in inhibiting tumor growth. However, utilizing CDK4/6 inhibitors as a standalone treatment often leads to the development of drug resistance. This resistance is thought to be linked to FAK signaling, which mediates CDK4/6-independent activation of CDK2, driving cell cycle progression and fostering cell survival even in the presence of CDK4/6 inhibitors (Jiang H et al., 2020). Furthermore, research suggests a correlation between CDK4/6 activity and the subcellular localization of FAK in B16F10 melanoma cells. Inhibiting FAK kinase activity promotes nuclear the localization of FAK. In its inactive state, nuclear FAK, leveraging its scaffold function, recruits CDH1 and CDK4/6 to its N-terminal FERM domain. This recruitment facilitates the ubiquitination and proteasomal degradation of CDK4/6, suppressing melanoma cell proliferation. Importantly, this process occurs exclusively when FAK is localized within the nucleus (Murphy et al., 2022). The scaffold structure of nuclear FAK has been observed in multiple studies to possess the ability to facilitate the degradation of various nuclear factors in multiple studies (Jeong et al., 2019; Zhou et al., 2019; Jeong et al., 2021; Jeong et al., 2022). Therefore, concurrent inhibition of FAK and CDK4/6 expression holds the potential to overcome drug resistance. Currently, preclinical studies in intrahepatic cholangiocarcinoma and diffuse gastric cancer (DGC) have demonstrated the synergistic anti-cancer effects of combining FAK and CDK4/6 inhibitors (Song et al., 2021; Peng et al., 2023) These findings provide a solid theoretical foundation for future clinical studies in this area.

### 3.2 FAK promotes tumor cell migration and invasion

The heightened expression of FAK is intricately linked with unfavorable outcomes for cancer patients due to its pivotal role in promoting tumor metastasis. FAK’s influence on cell migration involves its participation in the integration and resolution of components within the focal adhesion complex, coupled with dynamic interactions with intracellular Actin and the ECM. In migrating cells, the contraction of myosin stress fibers attached to the focal adhesion complex exerts force to regulate cell migration (Tapial et al., 2020; Le Coq et al., 2022). Additionally, various intracellular protein molecules have been identified that activate FAK, thereby promoting tumor cell invasion and migration (Hu et al., 2019; Jiang W et al., 2020; Dong et al., 2021; Kim et al., 2021). For example, PPFIA binding protein 1 induces the movement of glioblastoma U87 MG and U251 MG cell lines by interacting with FAK to activate Src and JNK (Dong et al., 2021). Similarly, Rho-associated protein kinase 1 (ROCK1) enhances the migratory ability of NSCLC cells through the PTEN/PI3K/FAK signaling pathway (Hu et al., 2019). Sialylation, a terminal glycosylation modification of glycoproteins, plays a crucial regulatory role in facilitating tumor cell adhesion and immune evasion (Jarahian et al., 2021; Pietrobono and Stecca, 2021). Sun et al. employed the CRISPR/Cas9 system to establish a stable FAK knockout (KO) cell line in HeLa cells, revealing that sialylation levels were significantly reduced in KO cells, leading to inhibited cell migration. Specifically, FAK primarily regulates N-glycan sialylation via the FAK/PI4KIIα/GolPH3/ST axis, reaffirming FAK’s unique position in multiple pathways regulating cell migration (Sun et al., 2023).

Epithelial-mesenchymal transition (EMT) plays a crucial role in the infiltration and spread of cancer cells, and FAK expression is positively correlated with EMT. Epidermal growth factor induces EMT in colorectal cancer cells by activating FAK (Huang et al., 2020). Tspan9 stimulates osteosarcoma migration by inducing EMT via activation of the FAK/Ras/ERK1/2 signaling cascade (Shao et al., 2022). The activated ERK further promotes cell contraction and stimulates tumor cell movement by driving actin polymerization and edge protrusion adhesion turnover (Samson et al., 2022). FAK has been identified as a significant regulatory factor for interleukin-6 induced EMT in colorectal cancer (Huang et al., 2023). In DGC, the loss of CDH1 (encoding E-cadherin, a key regulator of the EMT) and RHOA Y42C mutation in gastric organs of engineered mice co-activate the FAK/AKT/β-catenin and YAP-TAZ pathways, promoting the transformation of normal gastric epithelial cells into highly invasive DGC cells (Zhang et al., 2020). Despite the single drug resistance observed with FAK inhibitors, studies have shown that combining FAK inhibitors with MAPK inhibitors can effectively eliminate compensatory ERK activation, synergistically inhibiting the migration and invasion of malignant tumors such as DGC and UM (Paradis et al., 2021; Peng et al., 2023). Further investigation is warranted to uncover the specific molecular mechanisms underlying this phenomenon.

### 3.3 FAK regulates tumor angiogenesis

Angiogenesis is essential for the malignant development of tumors, and key regulatory molecules such as vascular endothelial growth factor (VEGF), VEGFA, and vascular endothelial growth factor receptor 2 (VEGFR2) play crucial roles in this process (Wang et al., 2020; Shiau et al., 2021; Patel et al., 2023). VEGFA, with a strong affinity for VEGFR2, promotes angiogenesis by activating downstream pathways, including the FAK-paxillin pathway. This activation facilitates the proliferation, survival, and migration of vascular endothelial cells (Simons et al., 2016; Wang et al., 2020). The VEGFR2-FAK signaling pathway induces VEGFA secretion, promoting angiogenesis and vascular permeability (Li L et al., 2022). In triple-negative breast cancer (TNBC), The results showed a positive correlation between FAK and VEGFR2 expression was observed, and knockout of FAK inhibited endodermal tube formation and angiogenesis in zebrafish, suppressing suppressed VEGF and VEGFR2 expression at the molecular level (Shiau et al., 2021).

Temporal quantitative phosphoproteomic analysis of human umbilical vein endothelial cells revealed that FAK phosphorylation activation is an early phosphorylation-dependent signaling event in the VEGFA/VEGFR2 pathway, emphasizing the crucial role of FAK in initiating angiogenesis (Abhinand et al., 2023). Notably, distinct phosphorylation sites on EC-FAK have divergent effects on tumor angiogenesis *in vivo*. EC Cre+; FAK Y397F^/Y397F^ small mutant mice exhibited constitutive reduction in tumor growth and angiogenesis, while EC Cre+; FAK Y861F^/Y861F^ mice showed normal tumor growth without significant inhibition of angiogenesis. These effects were attributed to decreased VEGFR2 expression, attenuated integrin β1 activation, and disruption of downstream FAK/Src/PI3K/AKT signaling induced by EC FAK-Y397F (Pedrosa et al., 2019). However, studies by Marina Roy-Luzarraga et al. found that inducing endothelial FAK deficiency in both orthotopic and spontaneous mouse model of pancreatic ductal adenocarcinoma (PDA) did not hinder angiogenesis but reduced the incidence of tumor metastasis and improved mouse survival (Roy-Luzarraga et al., 2022). However, Combining EC-FAK inhibition with other tumor therapies, such as Doxorubicin for melanoma (Tavora et al., 2014), demonstrated potential effectiveness in suppressing tumor angiogenesis, suggesting distinct effects of FAK inhibition when combined with different cancer types. The impact of EC-FAK on tumor angiogenesis may be intricately linked to the regulation of EC barrier function (Jean et al., 2014; Roy-Luzarraga et al., 2022).

Contrary to FAK expression in ECs, FAK expressed in pericytes (perivascular cells) may exert an opposing regulatory effect on tumor angiogenesis (Lechertier et al., 2020). The loss of FAK in pericytes enhances GAS6-stimulated receptor tyrosine kinase Axl phosphorylation and upregulates Cyr61, promoting tumor growth (Lechertier et al., 2020; Zhang et al., 2023b). Notably, FAK-Y861 in pericytes plays a pivotal regulatory role in tumor vascular regression and control of tumor growth (Lees et al., 2021). Targeting FAK specifically in ECs rather than pericytes remains an urgent challenge for future anti-FAK therapy.

## 4 Role of FAK in TME

**FIGURE 3:**
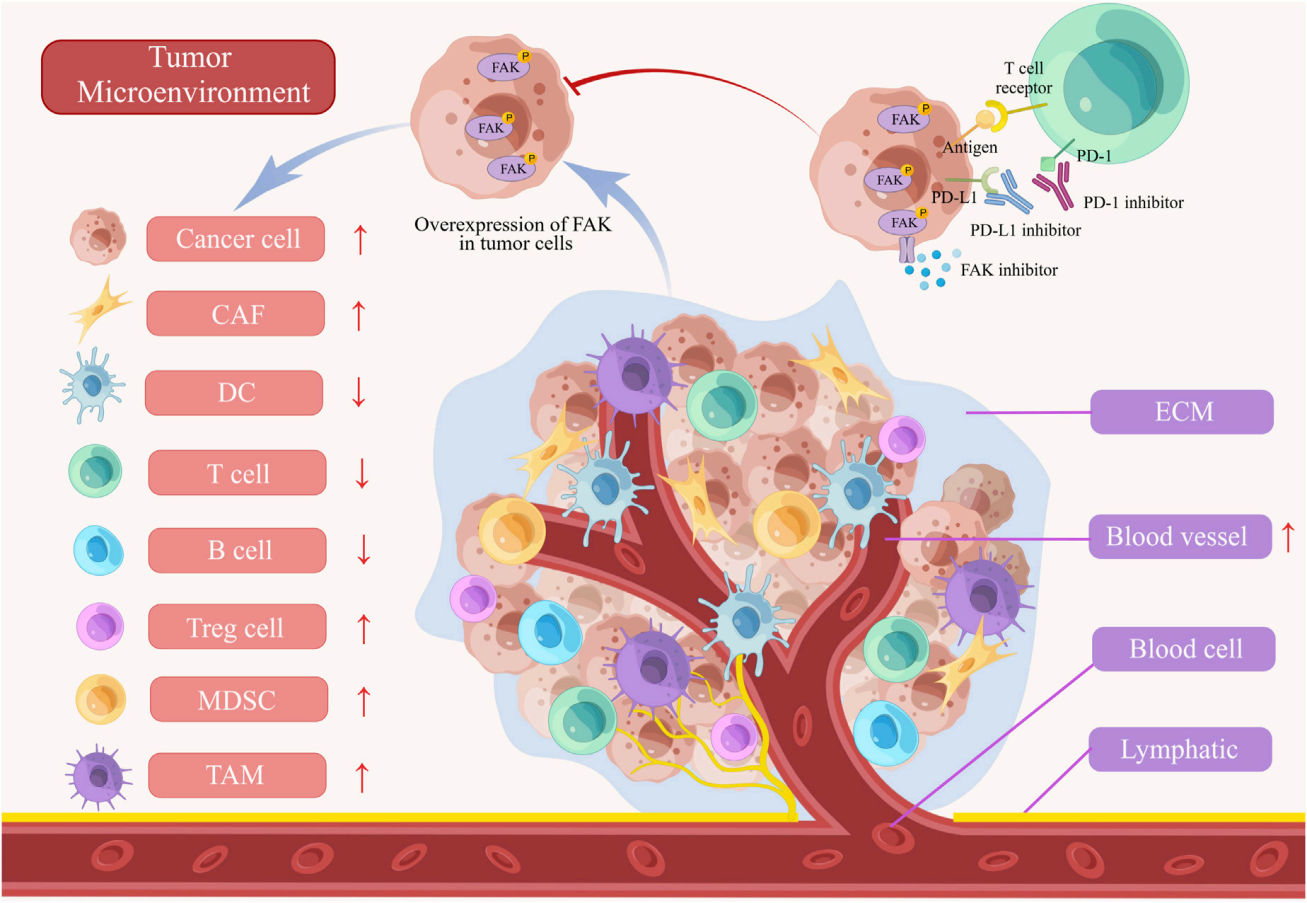
The role of FAK in tumor microenvironment (TME). The abnormal activation of FAK inhibits T cells, B cells, and dendritic cells (DCs) in the immune microenvironment. Furthermore, the activation of FAK leads to the promotion of myeloid-derived suppressor cells (MDSCs), tumor-associated macrophages (TAMs), cancer-associated fibroblasts (CAFs), and angiogenesis, all of which contribute to the progression of the tumors. FAK inhibitors, when combined with PD-1/PD-L1 inhibitors, demonstrate a more powerful anti-tumor effect by blocking tumor growth and enhancing immune cell functionality. ECM, extracellular matrix.

TME comprises a diverse array of cell types, including cancer cells, immune cells, dendritic cells, tumor-associated macrophages (TAMs), cancer-associated fibroblasts (CAFs), tumor blood vessels, lymphatics, adipocytes, and an ECM with collagen and elastin fibrous networks, along with numerous cytokines. This complex and dynamic ecosystem plays a pivotal role in evading immune responses and promoting tumor progression (Xiao and Yu, 2021). FAK influences various TME cell populations, ECM architecture, and associated signaling pathways involved in immunosuppression and matrix regulation, orchestrating the development of the immunosuppressive TME (Figure 3) (Osipov et al., 2019).

ECM is a crucial component in mediating FAK’s regulation of the TME. CAFs are key regulators of tumor occurrence and progression, residing in the tumor matrix surrounding tumor cells. CAFs promote tumor fibrosis, resisting various therapies by synthesizing collagen and other ECM molecules. Lumican, overexpressed in CAFs, activates the integrin β1/FAK pathway, promoting the growth and migration of gastric cancer cells (Wang et al., 2017). In PDA, CAFs play a pivotal role in promoting clonal growth, self-renewal, and migration, associated with enhanced differentiation activity of CSCs. Inhibiting FAK kinase activity in PDA cells eliminates the influence of CAFs on clonal growth (Begum et al., 2019). Lysyl oxidase-rich extracellular vesicles from CAFs promote tumor EMT through the activation of the p-FAK/p-axis/YAP pathway, identified as a pivotal step in collagen cross-linking. Targeting FAK disrupts this process (Liu X et al., 2023b). However, FAK-targeted therapy can lead to stroma depletion in the TME and decreased resident fibroblasts, resulting in reduced TGF-β secretion and weakened inhibition of the STAT3 signaling pathway. Combining FAK and STAT3 targeting alleviates this impact (Jiang H et al., 2020). Discoidin domain containing receptor 1 (DDR1), crucial for ECM stability through collagen interaction, has FAK as an important downstream regulatory molecule. Targeting DDR1 reshapes the TME, inhibiting chemotherapy resistance induced by the ECM in pancreatic cancer through the DDR1/PYK2/FAK pathway (Ko et al., 2022). Matrix stiffness induces lipid metabolic crosstalk between tumor and stromal cells, leading to bevacizumab resistance in colorectal cancer liver metastases. FAK, in the FAK/YAP pathway, plays a crucial role in this process. Inhibiting FAK enhances anti-VEGF therapy efficacy by suppressing hepatic stellate cell lipolysis (Zheng et al., 2023).

Despite the promise of immune checkpoint inhibitors, only a fraction of cancer patients benefits from them due to the immunosuppressive effects within the TME, involving regulatory T cells (Tregs), myeloid-derived suppressor cells (MDSCs), and TAMs. Inhibiting FAK expression reduces the presence of these immunosuppressive cells, affecting CSCs differentiation, and creating a more favorable TME for anti-tumor immune responses (Osipov et al., 2019). Furthermore, FAK has been found to inhibit the expression of PD-L1 on tumor cells, enhancing the recognition efficiency of cytotoxic T cells (Li et al., 2019; Zhang D et al., 2022). Positive correlations between PD-L1 and FAK was discovered in PD-L1-positive triple-negative breast cancer (TNBC) tissue samples (Mohan et al., 2019). The infiltration of various immune cells (CD8^+^ T cells, CAFs, and MDSCs) correlates with FAK expression. Blair et al. suggest that a promising strategy to modulate the immune microenvironment and enhance immunotherapy efficacy involves targeting stromal components in combination. Their study showcased the effectiveness of co-inhibition strategies targeting hyaluronic acid degradation and FAK, in combination with PD-1 blockade. This combined approach specifically reduced the number of granulocytes and bone marrow-derived cells expressing C-X-C chemokine receptor type 4 (CXCR4). Simultaneously, it facilitated T cell infiltration leading to an enhancement in the therapeutic outcomes of immune-based interventions for pancreatic cancer (Blair et al., 2022). Combining the FAK inhibitors with anti-PD-1antibodies demonstrated enhanced anti-cancer effects in C57BL/6 J primary hepatocellular carcinoma model with complete immune function, reducing Tregs and TAMs while increasing CD8^+^ T cell population (Wei et al., 2021). FAK also impedes antigen the processing and presentation in pancreatic cancer, contributing to immune evasion of pancreatic cancer. Depletion of FAK enhances anti-tumor activity by upregulating immune proteasome, MHC-I, and increasing CD8^+^ T cell infiltration (Blanco-Gomez and Jorgensen, 2023; Canel et al., 2023). CD11b, a protein in myeloid cells, enhances T cell-mediated immunity through downregulation of interferon gene expression, leading to suppression of tumor progression by decreasing infiltrating myeloid cells. Inhibiting FAK-mediated mitochondrial dysfunction activates the STING/STAT1 pathway, contributing to this process (Schmid et al., 2018; Panni et al., 2019; Liu Y et al., 2023a).

FAK serves as a potential target for radiotherapy, modulating tumor immune responses. A study demonstrated that radiation alone or in combination with checkpoint immunotherapy failed to elicit antigen-specific T-cell responses in PDA. In contrast, In the p48-Cre/LSL-KrasG12D/p53Flox/Flox (KPC) genetically engineered mouse models, combined administration of FAK inhibitors with checkpoint immunotherapy and radiation led to complete tumor regression and long-term survival in spontaneous PDA mice. This highlights FAK inhibition’s role in facilitating radiotherapy-induced tumor immunity and enhancing responsiveness to checkpoint immunotherapy (Lander et al., 2022). Additionally, combining low-dose radiotherapy with FAK inhibitors mitigated fibrosis and hypoxia in pancreatic cancer, promoting CD8+T cell infiltration and enhancing sensitivity to cancer radiotherapy (Chen H et al., 2022). Therefore, Clinical research is needed to assess the safety and efficacy of this combination therapy.

## 5 FAK mediates drug resistance

The pivotal role of FAK in regulating chemoradiotherapy resistance across various cancers underscores its potential as a therapeutic target. Targeting FAK has demonstrated sensitizing effects on both radiotherapy and various chemotherapy treatments (Allert et al., 2022; Gu et al., 2022; Jang et al., 2022; Li Y et al., 2022; Ling et al., 2022; Wang C et al., 2022; Yu et al., 2022; Gao et al., 2023). Most chemotherapy drugs, including platinum-based agents and fluoropyrimidine chemotherapy, exert their anticancer effects by inducing DNA damage in cancer cells (Newport et al., 2022). FAK, as a crucial regulatory protein in DNA damage repairs, orchestrates its regulatory function by facilitating the nuclear translocation of β-catenin. This process regulates the transcription of DNA damage repair genes, promoting cell survival and ultimately contributing to drug resistance. Targeting FAK emerges as a promising strategy to restore sensitivity to DNA damage therapy (Tavora et al., 2014; Newport et al., 2022; Roy-Luzarraga et al., 2022; Gao et al., 2023). Notably, Pifer et al. reported that targeting FAK suppresses homologous recombination and nonhomologous end-joining repair in p53-mutant HPV-negative head and neck squamous cell carcinoma cell lines, thereby enhancing the DNA-damaging effects of radiotherapy (Pifer et al., 2023). Additionally, in human CDH1-deficient cell line (SNU-668 and NUGC-4)-derived xenograft models in mice, ROS1 inhibitors activate the FAK-YAP-TRX signaling pathway, mitigating oxidative stress-induced DNA damage, thereby attenuating their anticancer efficacy (Gao et al., 2023).

Furthermore, FAK frequently facilitates the emergence of drug resistance in specific tumor types undergoing targeted therapy for particular gene mutations:1. BRAF/KRAS: Tumors harboring BRAF or KRAS mutations can effectively suppress the RAS/RAF/MEK/ERK pathway with BRAF or MEK inhibitors. However, tumor cells rapidly develop adaptive or acquired resistance mechanisms often accompanied by exhibit heightened expression of FAK and activation of the downstream Wnt/β-catenin signaling pathway (Fallahi-Sichani et al., 2017; Chen et al., 2018). This phenomenon may be related to the negative regulation of FAK signal transduction by the RAS/RAF/MEK pathway (Zheng et al., 2009). Additionally, CRISPR/Cas9 genome screening revealed significant enrichment of the Grb7 gene in KRAS mutant colorectal cancer cells exhibiting resistance to MEK inhibitors. This gene facilitates the activation of the FAK pathway through RTK signaling, triggering the ERK/MAPK signaling pathways and conferring resistance to MEK inhibition in tumor cells (Yu et al., 2022). 2. CDH1: CDH1-deficient tumors, characterized by a poor response to chemotherapy and increased susceptibility to drug resistance, often show upregulated FAK expression as a prognostic marker (Yuen et al., 2021). Treatment with FAK inhibitors has demonstrated significant efficacy against CDH1-deficient gastric cancer, characterized by downregulated E-cadherin and damaged membrane E-cadherin/β-catenin protein complexes a, resulting in reduced sensitivity of tumor cells to chemotherapy. The dense collagen matrix in gastric cancer cells further increases the interaction between integrin-mediated ECM and activated FAK/ERK signaling, facilitating the nuclear translocation of β-catenin and promoting the invasion and metastasis of tumor cells (Jang et al., 2018). In addition to CDH1 deficiency, DGC, often accompanied by RHOA gene mutation and activation of the YAP downstream signaling pathway, promotes tumor survival. FAK, a classic upstream regulator of YAP, inhibits the activation of the YAP pathway, restores the E-cadherin/β-catenin complex, and remodels ECM. Combining FAK inhibitors with chemotherapy enhances the anticancer effect synergistically (Gao et al., 2023). 3. HER2: FAK mediates resistance to anti-HER2 targeted drugs. The N-terminal FERM domain structure of FAK interacts with HER2, and the Src-FAK signaling pathway activates key downstream signaling pathways involved in HER2 crosstalk (Nahta, 2012). Activated FAK can further activate the downstream AKT, ERK and STAT3 pathways promoting drug resistance. Therefore, targeting FAK emerges as a promising strategy to overcome anti-HER2 drug resistance (Lazaro et al., 2013; Jin et al., 2017; Cooper and Giancotti, 2019; Castro-Guijarro et al., 2023). However, not all HER2-positive cancers exhibit sensitivity to FAK inhibition. Recent studies have demonstrated that HER2-positive breast cancer patients with elevated levels of circCDYL2 experience rapid recurrence after anti-HER2 therapy compared with those with lower levels of circCDYL2. Mechanistic investigations have revealed that circCDYL2 stabilizes Grb7 by preventing its ubiquitination and degradation, enhancing its interaction with FAK, and sustaining downstream AKT and ERK1/2 activity, mediating trastuzumab resistance. The application of FAK inhibitors has been shown potential in ameliorating trastuzumab resistance in cells exhibiting high levels of circCDYL2 (Ling et al., 2022). Future research should focus on screening specific tumor types highly sensitive to FAK inhibitors to explore their potential clinical application value.

The presence of CSCs plays a significant role in conferring drug resistance and promoting tumor recurrence. FAK, as a critical regulator of CSC activity, exerts a significant influence (Yin et al., 2021; Jang et al., 2022). CSCs rely on the highly expressed laminin to bind to integrin α6β1, promoting FAK-mediated self-renewal signaling of CSCs (Cooper and Giancotti, 2019). Yin et al. found that ATP-binding cassette subfamily G member 1 mediates the signaling of extracellular matrix protein-1-integrin αXβ2 interaction, leading to the activation of FAK/Rho/cytoskeleton molecules and conferring cisplatin resistance on cancer cells by upregulating CD326-mediated stemness (Yin et al., 2021). Moreover, the overexpression of KRT17 is correlated with unfavorable OS and reduced responsiveness to platinum-based therapy in patients diagnosed with oral squamous cell carcinoma The interaction between KRT17 and plectin (a macromolecular cytoskeletal protein) triggers the activation of the integrin β4/FAK/ERK pathway, thereby facilitating the stabilization and nuclear translocation of β-catenin while augmenting oral squamous cell carcinoma stemness and CD44 expression (Jang et al., 2022).

## 6 Development and clinical research progress of FAK inhibitors

**FIGURE 4:**
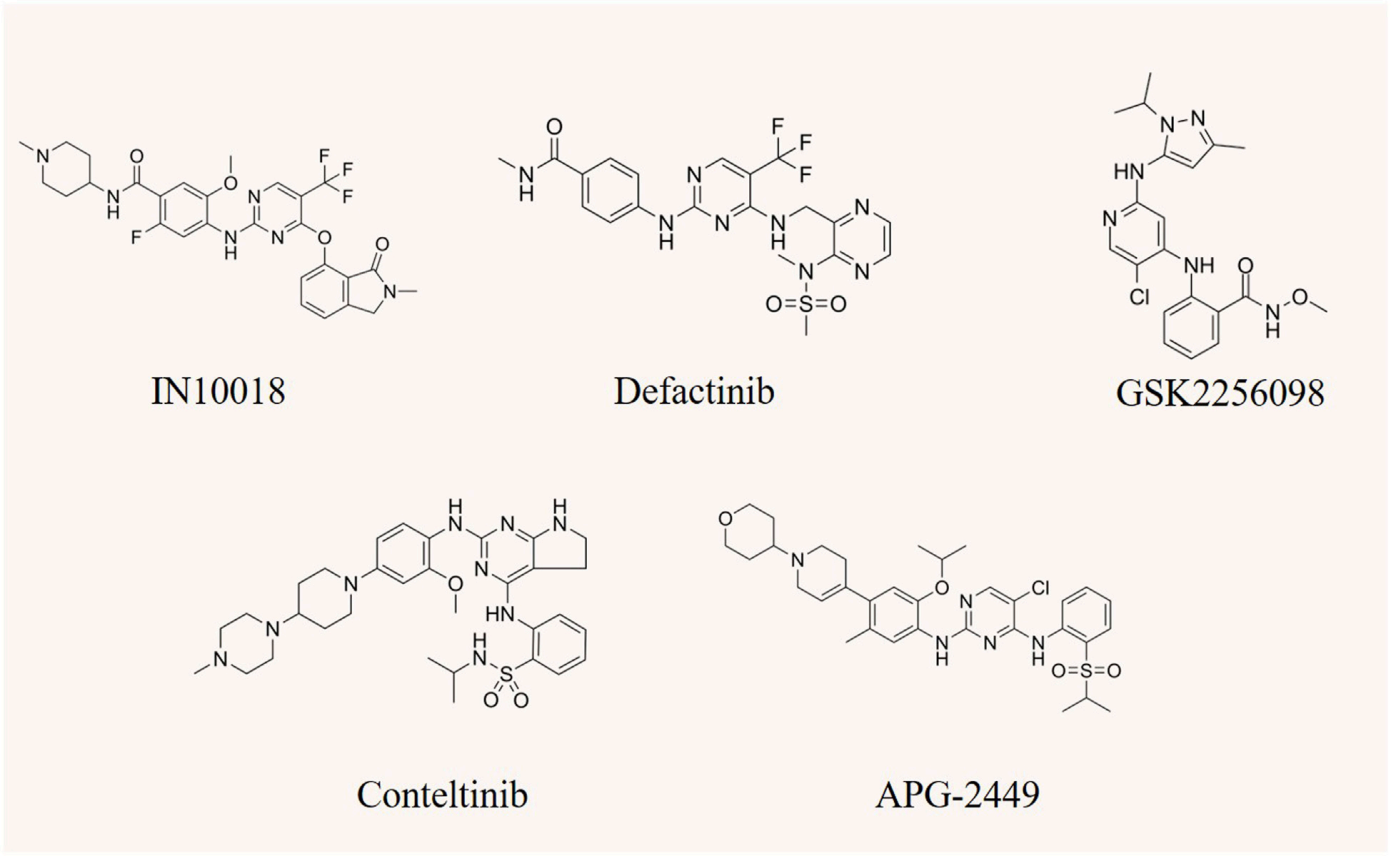
The chemical structure of FAK inhibitors IN10018, defactinib, GSK2256098, conteltinib, and APG-2449.

Targeting FAK has demonstrated efficacy in cancer therapy, particularly when standard treatments prove ineffective or in combination with other drugs. The ongoing development of novel FAK inhibitor reflects the significance of this approach. However, despite the active pursuit, no FAK inhibitor has yet received clinical approval. Most of them are currently in preclinical or clinical development stages (Quispe et al., 2022). FAK inhibitors can be categorized into allosteric site inhibition, ATP-competitive inhibition of kinase inhibitors, as well as FERM domain and FAT domain inhibitors. Notably, only ATP-competitive inhibitors of FAK have advanced to the clinical research stage (Mustafa et al., 2021b; Spallarossa et al., 2022). Consequently, this article will focus on providing a detailed overview of representative drugs within this class, covering their progress in both preclinical and clinical studies (Table 1). Certain studies within this class have shown promising advancements, instilling new hope for patients who are responsive to FAK inhibition therapy (Figure 4). Additionally, recent developments in FAK degraders based on PROTAC technology have addressed the limitation of FAK scaffold function being untargetable by small molecule inhibitors (Huo et al., 2022). This breakthrough opens avenues for exploring novel mechanisms of FAK degradation, aiming to enhance anticancer efficacy and gain new insights into targeted FAK therapy. The evolving landscape of FAK inhibitors, coupled with innovative approaches like PROTAC technology, holds the potential to reshape cancer therapy. Ongoing research endeavors are critical for advancing our understanding and application of these promising treatments.

**TABLE 1:** Summary of FAK inhibitors in clinical studies.

Drug	Target	Tumor type	Clinical stage	NCT Trial No.	References
IN10018	FAK	Metastatic non-hematologic malignancy	I	NCT01335269	de Jonge et al. (2019)
IN10018	FAK	Advanced or metastatic solid tumors	I	NCT01905111	Doi et al. (2019)
IN10018 +PLD	FAK	Platinum-resistant ovarian cancer	I/II	NCT05551507	Wu X et al. (2022)
Defactinib	FAK/PYK2	Merlin protein low expression epithelioid sarcoma	II	NCT01870609	Fennell et al. (2019)
Defactinib	FAK/PYK2	KRAS mutant NSCLC	II	NCT01951690	Gerber et al. (2020)
Defactinib+ Pembrolizumab + Gemcitabine	FAK/PYK2 PD-1	Intractable pancreatic cancer	I	NCT02546531	Wang-Gillam et al. (2022)
Defactinib+ VS-6766	FAK/PYK2RAF/MEK	KRAS mutant NSCLC	II	NCT04620330	Capelletto et al. (2022)
GSK2256098	FAK	Advanced solid tumor	I	NCT01138033	Soria et al. (2016)
GSK2256098+ Trametinib	FAK MEK	Advanced solid tumor	Ib	NCT01938443	Mak et al. (2019)
GSK2256098	FAK	NF2 Mutant Meningioma	II	NCT02523014	Brastianos et al. (2023)
Conteltinib	FAK/PYK2/ALK	Advanced ALK-positive NSCLC	I	NCT02695550	Xing et al. (2022)
APG-2449	FAK/PYK2/ALK	Advanced solid tumor	Ib/II	NCT03917043	Zhao et al. (2022)

Note: FAK, focal adhesion kinase; PLD, polyethylene glycol-conjugated liposomal doxorubicin; NF2, neurofibromatosis type 2; PYK2, protein tyrosine kinase; NSCLC, non-small cell lung cancer.

### 6.1 IN10018 (BI 853520; Ifebemtinib)

IN10018 stands out as a highly efficient and selective ATP-competitive FAK inhibitor. Biomarker analysis and gene set enrichment studies have unveiled a heightened sensitivity of IN10018, particularly associated with the mesenchymal tumor phenotype. This sensitivity is notably correlated with elevated E-cadherin expression. Impressively, IN10018 demonstrates rapid and persistent inhibition of FAK autophosphorylation in the tumor tissue of immunodeficient mice (Hirt et al., 2018; Tiede et al., 2018). IN10018 shows robust anti-cancer activity both in breast cancer cells *in vitro* and multiple preclinical mouse models. It also inhibits the *in vitro* growth of malignant pleural mesothelioma cell spheroids, significantly reduces tumor weight in mice, and show effective inhibitory effects on cell proliferation and microvascular growth in tumor tissue (Laszlo et al., 2019). IN10018 also inhibit EMT and tumor growth *in vivo* of ovarian cancer cells through the FAK/AKT/mTOR signaling pathway (Li et al., 2021). Moreover, in KPC orthotopic murine model, IN10018 enhances the sensitiveness of PDA to radiotherapy. Compared with single radiotherapy or FAK inhibition therapy, the combination of FAK inhibitor and radiotherapy significantly increases the infiltration of CD8^+^ T cells and macrophages (Osipov et al., 2021).

The initial findings from the phase I study of IN10018 in human subjects demonstrate its safety and excellent tolerability among advanced non-hematologic malignancy patients. The maximum tolerated dose (MTD) was determined to be 200 mg daily. Out of the 63 patients enrolled, 49 were evaluable, and 17 (27%) achieved disease stabilization as the best response, with four cases exhibiting stability for over 150 days (de Jonge et al., 2019). Additionally, a phase I research conducted with patients having advanced or metastatic solid tumors in Japan and Taiwan revealed favorable pharmacokinetics and safety profiles for IN10018. The median disease control duration was recorded at 3.7 months, indicating promising anti-tumor activity (Doi et al., 2019). The prospect of combination therapy involving IN10018 appears to be a feasible approach for maximizing the anti-tumor effects of FAK inhibitors. A phase I study of IN10018 combined with pegylated liposomal doxorubicin for platinum-resistant ovarian cancer showed an ORR of 56.7% (95% CI: 37.4%–74.5%) and a DCR of 86.7% (95% CI: 69.3%–96.2%) in the 30 evaluable patients. The observed median progression-free survival (PFS) among all 42 enrolled patients was 6.2 months (95% CI: 6.2 months–NA), suggesting that the combination of IN10018 and pegylated liposomal doxorubicin has elucidated therapeutic effects and manageable safety in platinum-resistant ovarian cancer patients (Wu X et al., 2022).

### 6.2 Defactinib (VS-6063)

Defactinib is a highly efficient and reversible dual inhibitor of FAK and Proline-rich tyrosine kinase 2 (PYK2), belonging to the class of ATP-competitive inhibitors. Defactinib inhibits the phosphorylation of FAK Tyr397 in a time- and dose-dependent manner. Many studies have demonstrated that defactinib effectively inhibits in various types of FAK-overexpressing cancers by effectively blocking the PI3K/AKT and downstream signaling (Zhang B Y et al., 2021; Cuellar-Vite et al., 2022; Liu et al., 2024). Moreover, a negative correlation was found between FAK activation and the sensitivity of breast cancer cells to rapamycin. In preclinical models, the inhibition of FAK has shown the potential to increase the sensitivity of rapamycin-resistant tumors to mTORC1 inhibition, suggesting that targeting FAK signaling could be a feasible and effective strategy to enhance the efficacy of mTORC1 inhibitors in resistant cancers (Cuellar-Vite et al., 2022). Moreover, the combination of docetaxel and defactinib has demonstrated significant reductions in the survival rate of docetaxel-resistant prostate cancer cells *in vitro*. Additionally, it effectively inhibits the growth of PC3 xenograft tumors. Notably, FAK expression is positively correlated with advanced tumor stages in human primary prostate cancer (Lin et al., 2018). Defactinib, when combined with osimertinib (an EGFR inhibitor), synergistically inhibits the activation of AKT and induces apoptosis in NSCLC. This combined therapy exhibits a higher therapeutic effect *in vivo* compared to single-drug therapy, suggesting a feasible strategy to overcome drug resistance of NSCLC to EGFR-TKI (Tong et al., 2019). Le Large et al. (Le Large et al., 2021) confirmed that defactinib has anti-proliferative and anti-migratory effects in PDA with overexpressed FAK. When combined with albumin-bound paclitaxel, it synergistically inhibits cell proliferation both *in vitro* and *in vivo*. Uterine serous carcinoma (USC), a distinct subtype of endometrial cancer with higher malignant potential than endometrioid endometrial carcinoma, exhibits enhanced oxidative stress compared with endometrioid endometrial carcinoma tumors. This heightened oxidative stress leads to the phosphorylation of FAK, facilitating tumor invasion and metastasis through the ROS-FAK-PAX signaling pathway. Defactinib significantly inhibited the growth of the tumors of patient-derived orthotopic xenograft models in this context, emphasizing the potential of FAK inhibition in the treatment of USC (Lopez-Mejia et al., 2023).

A phase II trial of defactinib for low Merlin protein-expressing malignant pleural mesothelioma did not demonstrate improvement in PFS and OS compared with placebo after first-line chemotherapy (Fennell et al., 2019). Consequently, it is not recommended to use defactinib alone as maintenance therapy for advanced malignant pleural mesothelioma. However, a phase I study for previously treated advanced KRAS-mutant NSCLC showed that defactinib monotherapy had good overall tolerability, moderate clinical activity, and efficacy in heavily pretreated patients with KRAS-mutant NSCLC, irrespective of TP53 and CDKN2A status (Gerber et al., 2020). This suggests heterogeneity in the response to FAK inhibition across different cancers. In addition, a phase I study revealed that the combination of defactinib, pembrolizumab, and gemcitabine was well-tolerated and did not exhibit any dose-limiting toxicity in patients with refractory pancreatic cancer. The disease control rate (DCR) of 20 evaluable patients with refractory pancreatic cancer was 80%, with the median PFS of 3.6 months and OS of 7.8 months. The combination regimen showed good tolerability and safety, demonstrated preliminary efficacy, and increased the infiltration of T lymphocytes in tumors (Wang-Gillam et al., 2022). Currently, a phase II clinical study known as RAMP-202 aims to assess the effectiveness and safety of the combination treatment of VS-6766 (an RAF/MEK inhibitor) and defactinib in patients with advanced KRAS-mutant NSCLC who have experienced treatment failure with previous platinum chemotherapy and immunotherapy (Capelletto et al., 2022).

### 6.3 GSK2256098

GSK2256098 is an orally available small molecule compound that exhibits high selectivity in inhibiting FAK to block adhesion, proliferation, and migration of cancer cells (Auger et al., 2012). A preclinical study indicated that GSK2256098 effectively blocks the FAK signaling pathway mediated by CPNE8 in gastric cancer cell migration and metastasis (Zhang P et al., 2022). Additionally, it inhibits the proliferation of various PDA cells (Zhang et al., 2014). The efficacy of GSK2256098 is closely associated with the abnormal expression of certain genes or proteins. Research indicates that PTEN-mutant endometrial cancer patients exhibit markedly superior responses to GSK2256098 treatment compared with patients with PTEN-wild type endometrial cancer (Thanapprapasr et al., 2015). In a phase I trial involving late-stage solid tumor patients, GSK2256098 demonstrated a controllable safety profile, with most adverse reactions ranking grade 1–2, and the maximum tolerated dose (MTD) was determined to be 1000 mg twice daily. When targeting at or below MTD doses, GSK2256098 exhibits clinical activity in mesothelioma patients, especially those with Merlin protein deficiency (Soria et al., 2016). Another phase Ib trial showed that the combined use of trametinib (a MEK inhibitor) and GSK2256098 to treat solid tumors also demonstrated good safety, supporting the progress of further clinical research (Mak et al., 2019). As NF2 mutations serve as malignant markers of meningioma, FAK inhibition, and NF2 loss have been shown to have a synthetic lethality relationship. A phase II trial examined the effectiveness of GSK2256098 in the treatment of patients with NF2 mutant meningiomas. The results demonstrated that GSK2256098 improved 6-month PFS in patients with recurrent or progressive NF2 mutant meningioma compared with that in the control group (Brastianos et al., 2023). These findings suggest the need for further evaluation of GSK2256098 in this specific patient population.

### 6.4 Conteltinib (CT-707; SY-707)

Conteltinib is a multi-kinase inhibitor targeting FAK, PYK2, and anaplastic lymphoma kinase (ALK). Initially developed as an ALK inhibitor due to its strong inhibitory activity against ALK (IC_50_ = 2.4 nM), conteltinib demonstrated manageable safety, favorable pharmacokinetic properties, and anticancer effects in patients with advanced ALK-positive NSCLC in a phase I human clinical study (Xing et al., 2022). In a breast cancer mouse model, conteltinib was found to inhibit tumor growth and spontaneous metastasis to the lung (Liu P et al., 2022), with the inhibitory effect on tumor invasion and metastasis primarily attributed to FAK inhibition. Preclinical studies further support conteltinib’s efficacy in inhibiting FAK kinase activity and blocking downstream signaling in various tumors, including liver and lung cancer. In liver cancer, conteltinib inhibits the growth of cancer cells *in vitro* and xenografts derived from patients *in vivo* by blocking the IGF1R-YAP signaling axis activated by hypoxia (Zhu et al., 2018). Additionally, conteltinib overcomes sorafenib resistance in hypoxia-mediated liver cancer by inhibiting the YAP signaling pathway (Chen Y et al., 2022). Moreover, conteltinib synergizes with cabozantinib, a MET inhibitor, to inhibit the progression of liver cancer by blocking cabozantinib-induced FAK inactivation (Wang et al., 2016). This approach with FAK-containing kinase inhibitors offers a new therapeutic strategy for refractory metastatic cancers. Previous studies have highlighted the role of FAK inhibition in enhancing immune surveillance by overcoming fibrosis and improving immunosuppressive TME in KPC mouse models. This enhancement results in an improved response of PDA to T cell immunotherapy and PD-1 antagonists (Jiang et al., 2016; Blair et al., 2022). Building on this, a Phase Ib/II, open-label, dose-escalation, and dose-expansion study has been initiated to evaluate the safety, tolerability, pharmacokinetics, and antitumor activity of conteltinib in combination with toripalimab (anti- PD-1 monoclonal antibody) and gemcitabine in advanced pancreatic cancer (NCT05580445).

### 6.5 APG-2449

APG-2449 is a multi-kinase inhibitor targeting FAK, ALK, and ROS1 in various cancers. In esophageal squamous cell carcinoma, the combination of APG-2449 and ibrutinib effectively inhibits the survival and invasion of cancer cells, inducing cell cycle arrest in the G1/S phase and promoting apoptosis. Mechanistically, this combination therapy significantly reduces the phosphorylation of MEK/ERK and AKT (Luo et al., 2021). Furthermore, APG-2449 has demonstrated the ability to sensitize drug-resistant ovarian xenograft tumors to carboplatin and paclitaxel. The inhibitory effect of APG-2449 on FAK activity contributes to a reduction in CD44-positive and aldehyde dehydrogenase 1-positive CSCs within the TME, thereby improving drug resistance (Fang et al., 2022). Currently, a phase I clinical trial (NCT03917043) is underway to evaluate the safety and preliminary efficacy of APG-2449 in advanced solid tumors (Zhao et al., 2022).

### 6.6 Other FAK inhibitors

In addition to the aforementioned FAK inhibitors, several drugs entered clinical research based on promising anti-tumor activity in preclinical studies. Unfortunately, these candidates failed to demonstrate significant improvements in disease treatment during subsequent clinical trials. Examples include PF-00562271 (NCT00666926), VS-4718 (NCT01849744; NCT02651727), and CEP-37440 (NCT01922752). Additionally, numerous FAK-targeted drugs are still in preclinical research, such as BJG-03-025 (Groendyke et al., 2021), PF-573228 (Slack-Davis et al., 2007), and Y15 (Hochwald et al., 2009) (Table 2), demanding extensive preclinical research data to support their transition to clinical application.

**TABLE 2:** Summary of FAK inhibitors in preclinical studies.

Drug	Target	CAS number	IC_50_ to FAK	Start time	References
BJG-03-025	FAK	2553213-90-2	20.2 nM	2020	Groendyke et al. (2021)
PF-573228	FAK	869288-64-2	4 nM	2007	Slack-Davis et al. (2007)
Y15	FAK	4506-66-5	-	2009	Hochwald et al. (2009)
PF-562271	FAK/PYK2	717907-75-0	1.5 nM	2008	Roberts et al. (2008)
FAK-IN-9	FAK	2911655-93-9	27.44 nM	2023	Zhang et al. (2023a)
PROTAC FAK degrader 1	FAK	2301916-69-6	6.5 nM	2018	Cromm et al. (2018)
NVP-TAE 226	FAK/IGF-1R	761437-28-9	5.5 nM	2007	Liu et al. (2007)
GSK215	FAK	2743427-26-9	-	2021	Law et al. (2021)
BI-3663	FAK/PYK2	2341740-84-7	18 nM	2019	Popow et al. (2019)
BI-4464	FAK	1227948-02-8	17 nM	2019	Popow et al. (2019)
FAK inhibitor 2	FAK	2354405-14-2	0.07 nM	2019	Su et al. (2019)
FAK-IN-7	FAK	19948-85-7	11.72 μM	2012	Yang et al. (2012)
FAK-IN-8	FAK	1374959-91-7	5.32 μM	2012	Yang et al. (2012)
FAK-IN-5	FAK	2408317-70-2	-	2020	Jorda et al. (2020)
FAK inhibitor 5	FAK	2237234-47-6	0.6 nM	2013	Iwatani et al. (2013)
FAK-IN-3	FAK	2882094-29-1	-	2022	Wei et al. (2022)
FAK-IN-4	FAK	-	-	2022	Yang L et al. (2022)
EGFR-IN-46	EGFR/FAK	2764772-88-3	14.25 nM	2022	Elbadawi et al. (2022)
FAK-IN-2	FAK	2872588-02-6	35 nM	2021	Chen et al. (2021)

Note: CAS, chemical abstracts service; FAK, focal adhesion kinase; PYK2, proline-rich tyrosine kinase 2; IGF-1R, insulin-like growth factor 1 receptor; EGFR, epidermal growth factor receptor.

Given the intricate crosstalk between FAK and multiple signaling proteins in tumor development, various dual or multiple inhibitors emerged to simultaneously target FAK and other pathways. Examples include HDAC2/FAK inhibitors (Compounds 6a (Mustafa et al., 2021a)), EGFR/FAK inhibitors (2-Arylquinolines (Elbadawi et al., 2022)), FAK/IGF-1R inhibitors (TAE-226 (Schultze et al., 2010) and INT2-31167 (Ucar et al., 2012)), and ALK/IGF-R1/FAK inhibitors (Certinib (Mehta et al., 2022)), FAK/ALK inhibitors (CEP-37440 (Ott et al., 2016)), and FAK/CDK4/6 inhibitors (Compounds 1–7 (Sun et al., 2021)). While most inhibitors have displayed promising anticancer activity in preclinical studies, certain drugs faced setbacks in further clinical investigation due to adverse effects (Wu L Y et al., 2022). Notably, TAE-226 remains in the preclinical research stage due to side effects causing severe dysregulation of glucose metabolism and blood glucose in animal models (Kurio et al., 2011). Recent preclinical studies have highlighted novel FAK inhibitors based on the lead compound TAE-226, such as 4-arylamino-pyrimidine derivatives (Long et al., 2023) and 2,4-diaminopyrimidine cinnamyl derivatives (Liu Y et al., 2023b). These compounds exhibit remarkable drug stability and potent anticancer activity, emphasizing the imperative need for further optimization of molecular structures to enhance drug safety, and stability, and reduce off-target effects for clinical application.

### 6.7 FAK degraders based on PROTAC

**FIGURE 5:**
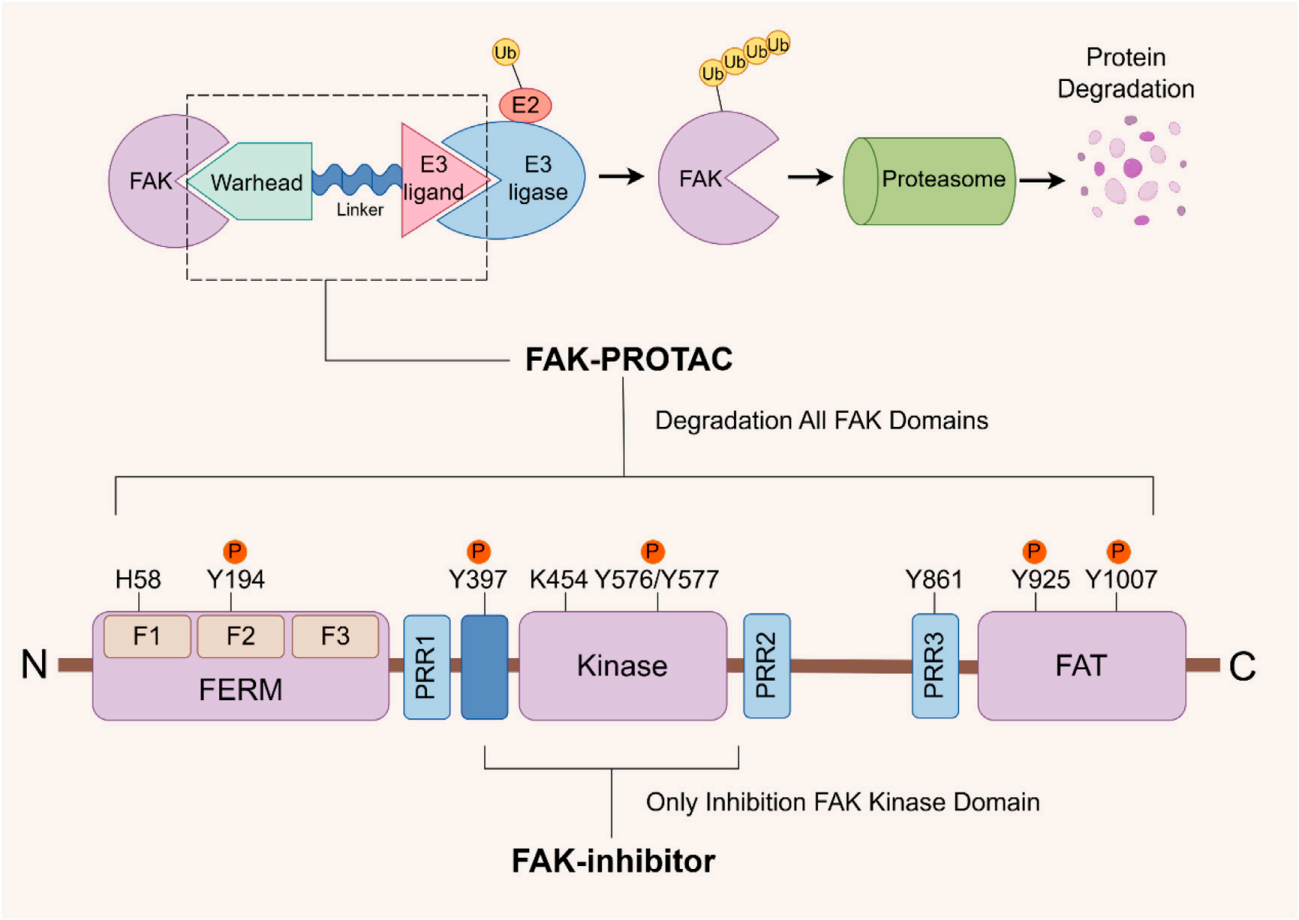
FAK- PROTAC domain structure and working principle. The FAK-PROTAC system comprises three essential components: a specifically designed warhead to bind to FAK, an E3 ubiquitin ligand responsible for recruiting E3 ubiquitin ligase, and a linker connecting the two. PROTAC functions by facilitating the proximity between FAK and E3 ubiquitin ligase, which subsequently leads to ubiquitination, followed by proteasome degradation. PROTAC: Proteolysis Targeting Chimera.

The successful development of FAK degraders through Proteolysis Targeting Chimera (PROTAC) technology has also opened up a new pathway for FAK targeted therapy (Pang et al., 2021). A PROTAC comprises three components: a warhead designed for specific binding to target proteins, an E3 ubiquitin ligand to recruit E3 ubiquitin ligases, and a linker to connect them. Specifically, PROTAC facilitates the proximity of the target protein with the E3 ubiquitin ligase, leading to ubiquitination and subsequent degradation through the proteasome system (Bekes et al., 2022). The primary distinction in FAK-PROTAC design lies in the variation of their ligands. The activity of PROTACs is closely associated with the expression of ligase RNA, DNA copy number, and protein levels (Luo et al., 2022). Currently, cereblon (CRBN) and Von Hippel-Lindau (VHL)-based PROTACs are extensively utilized due to their low molecular weight, favorable drug formation, and facile synthesis (Luo et al., 2022; Jiang et al., 2023). Utilizing PROTAC technology, a diverse range of degraders targeting androgen receptor (AR), estrogen receptor (ER), Bruton’s tyrosine kinase (BTK), STAT3, BRD4, and other protein molecules have been developed. While certain drugs like ARV-110 (targeting AR) and ARV-471 (targeting ER) have exhibited remarkable efficacy in clinical trials, the degraders targeting FAK are yet to progress into the clinical stage (Chen Z et al., 2022; Liu Z et al., 2022; Zhao et al., 2023). Compared with the conventional small molecule inhibitors that solely inhibit kinase activity, PROTAC technology effectively eliminates both kinase-dependent enzymatic activity and scaffold function by inducing degradation of FAK (Figure 5). This approach circumvents drug resistance arising from the restoration of FAK functionality (Huo et al., 2022).

Cromm et alprepared PROTAC 3, a compound that efficiently degrades FAK at low concentrations, inhibiting 95% of Fak at 50 nM. This compound is created by conjugating a modified defactinib warhead to a VHL ligand via a linker. Their study findings demonstrate the superiority of PROTAC 3 over the FAK inhibitors defactinib in terms of activating FAK and inhibiting downstream proteins paxillin and AKT signaling (Cromm et al., 2018). Notably, both FAK encoding genes PTK2 and ASAP1 are located in the oncogenomic locus 8q24 and are associated with tumor metastasis and recurrence. By blocking FAK kinase activity and the interaction between FAK and ASAP1, PROTAC 3 exhibits high efficacy in inhibiting invasion and metastasis of ovarian cancer cells (Huo et al., 2022). GSK215 is a FAK highly selective PROTAC based on VHL E3 ligase adhesive and FAK inhibitor VS-4718. In mice, a single dose of GSK215 induced rapid and long-term degradation of FAK, having a lasting effect on FAK levels lasting approximately 96 h (Law et al., 2021). Additionally, another team developed and compared the consequences and advantages of the FAK degrader BSJ-04146 and the FAK inhibitor BSJ-04-175 in eliminating all FAK activities in cancer models. The results showed that, compared with kinase inhibition, the targeted degradation of FAK performed better in downstream signal transduction and cancer cell viability and migration (Koide et al., 2023). Based on the above findings, FAK-targeted PROTAC may emerge as a more promising research and development strategy, as well as a treatment modality, compared with FAK small molecular inhibitors in the foreseeable future.

In preclinical studies of lung cancer, two novel FAK-PROTACs (PROTAC-A13 and PROTAC B5) demonstrated superior FAK degradation compared with the FAK inhibitor PF-562271 (PROTAC-A13: 85% degradation at 10 nM; PROTAC-B5: 86.4% degradation at 10 nM), as well as potent anti-cancer activity (PROTAC-A13: IC50 value of 26.4 nM; PROTAC-B5: IC50 = 0.14 μM). At the same time, they exhibit excellent plasma stability and appropriate membrane permeability (Qin et al., 2022; Sun et al., 2022). Additionally, Professor Rao Yu’s team developed a FAK PROTAC (FC-11) based on the CRBN ligand, which exhibiting remarkable degradation activity with a DC50 value of 310 pM (Gao et al., 2020b). Furthermore, the team investigated the practical application potential and side effects of this compound and discovered that after treatment with FC-11, there was a significant decrease in both sperm count and vitality in the mouse epididymis. However, no impact on the reproductive system was observed when using the FAK inhibitor PF562271. Importantly, discontinuing the administration of PF562271 restored sperm vitality (Gao et al., 2020a). These findings offer promising prospects for the future development of reversible male contraceptives.

## 7 Summary and prospect

FAK is frequently overexpressed in various cancer types and is associated with poor prognoses for cancer patients. FAK plays a crucial role in mediating signaling pathways such as p53, RAS/RAF/MEK, and YAP/TAZ, promoting tumor cell survival and progression. Additionally, FAK influences the tumor immune microenvironment, affecting the expression and chemotaxis of immune cells and modulating ECM density to promote metastasis and drug resistance. As a result, FAK has become an attractive therapeutic target. Numerous small molecular inhibitors of FAK have been developed, some progressing to the clinical research stage. While FAK kinase function inhibitors have demonstrated safety and efficacy, there are currently no clinically approved FAK inhibitors. Recently, the focus has shifted to the development of FAK degradation agents based on PROTAC technology (Qin et al., 2022; Sun et al., 2022; Koide et al., 2023). This approach aims to induce the complete loss of FAK function through ubiquitination, reducing the occurrence of drug resistance. Nevertheless, the improvement of both high selectivity and specificity of FAK degraders remains a primary focus for future research and development endeavors. Additionally, the CRISPR/Cas9 system, comprising a small guide RNA and a functional Cas9 endonuclease protein, serves as a potent gene editing tool capable of precisely disrupting or modifying FAK at the DNA level. Simultaneously, other genes such as MEK can be targeted for knockout to investigate potential synergistic effects between related genes, offering promisingly exploration and application prospects (Paradis et al., 2021; Sun et al., 2023).

Current research indicates that FAK inhibitors show limited efficacy as monotherapy in cancer treatment, but promising results emerge when combined with other drugs. Additionally, while explaining the role of FAK in signaling pathways and tumor development, we found promising therapeutic targets that can be synergistically combined with FAK. Specifically, significant synergies were noted when FAK inhibitors were combined with mTOR inhibitors (Shi et al., 2016; Cuellar-Vite et al., 2022), RAF/MEK inhibitors (Capelletto et al., 2022; Gu et al., 2022; Tarin et al., 2023), CDK4/6 inhibitors (Murphy et al., 2022; Peng et al., 2023), MAPK inhibitors (Peng et al., 2023), and VEGF inhibitors such as bevacizumab (Zheng et al., 2023). Furthermore, we observed an upregulation of FAK in specific tumor types with gene mutations or aberrant protein expression, such as BRAF/KRAS mutations, CDH1 deletions, EGFR or HER2 overexpression, and mediated drug resistance processes (Tong et al., 2019; Yuen et al., 2021; Yu et al., 2022; Castro-Guijarro et al., 2023). Therefore, identifying tumor types that exhibit increased sensitivity to FAK is important for enhancing targeted efficacy and screening specific populations responsive to FAK inhibition. Inhibition of FAK enhances the sensitivity to chemotherapy drugs or radiotherapy by modulating DNA damage repair genes (Tang et al., 2016), making it a potential ally for immunotherapy by reshaping the TME. Combination therapy with FAK inhibitors is considered a promising treatment strategy with broad research prospects. Ongoing clinical studies on FAK inhibitors are awaited to provide further guidance for current research strategies targeting FAK.
